# Circulating Inflammatory Mediators and Genetic Polymorphisms of Inflammation Mediators and Their Association with Factors Related to Abdominal Aortic Aneurysm: A Systemic Review and Meta-Analysis

**DOI:** 10.31083/j.rcm2308270

**Published:** 2022-07-25

**Authors:** Hecheng Wang, Zhenwu Zhong, Deying Jiang, Hao Zhang, Fanxing Yin, Panpan Guo, Junyu Chen, Xinyu Zhu, Kui You, Yanshuo Han, Kun Liu

**Affiliations:** ^1^School of Life and Pharmaceutical Science, Dalian University of Technology, 124221 Panjin, Liaoning, China; ^2^Department of Vascular Surgery, Dalian Municipal Central Hospital, 116003 Dalian, Liaoning, China; ^3^School of Ocean Science and Technology, Dalian University of Technology, 124221 Panjin, Liaoning, China; ^4^Department of Cardiac Surgery, Affiliated Hospital of Guizhou Medical University, 550001 Guiyang, Guizhou, China

**Keywords:** abdominal aortic aneurysm, CRP, IL-6, IL-10, TNF-α, inflammatory mediators, single nucleotide polymorphism

## Abstract

**Background:**

This study aimed to explore the levels of circulating inflammatory factors CRP, IL-6, IL-10 and TNF-α based on the literature review. This study also examined the influence of single nucleotide polymorphism (SNP) sites on the susceptibility of abdominal aortic aneurysm (AAA) using meta-analysis and intended to provide additional information on pathogenesis of AAA research.

**Methods:**

Electronic databases including PubMed and Web of Science were systemically searched to collect the information on AAA, inflammatory factors such as CRP, IL-6, IL-10, TNF-α and the SNP sites for data extraction. Altogether six SNPs in four genes (rs3091244, CRP; rs1800947, CRP; rs1205, CRP; rs1800795, IL-6; rs1800896, IL-10; and rs1800629, TNF) were assessed.

**Results:**

This study enrolled altogether 41 relevant investigations involving 9,007 AAA patients to carry out meta-analysis. According to pooled analysis, circulating CRP and IL-6 levels were shown to be related to the AAA, while plasma IL-10 and TNF-α levels were not associated with AAA. The circulating CRP level standard mean difference (SMD) was 0.30 (95% confidence interval (CI): 0.17–0.43), the IL-6 level SMD was 0.34 (95% CI: 0.20–0.49), the IL-10 level SMD was –0.01 (95% CI: –0.09–0.06), and the TNF-α level SMD was 0.09 (95% CI: 0.00–0.19). Similarly, the odds ratio (OR) of rs3091244 (CRP) under the recessive gene model was 1.70 (95% CI: 1.13–2.57). In addition, individuals with A and T mutant genes at locus rs3091244 might have a higher tendency of AAA susceptibility than those with C allele. Consecutively, the OR was 0.91 (95% CI: 0.51–0.97) for rs1800795 (IL-6) locus in the allele model, and individuals with G mutant gene at locus rs1800795 (IL-6) might be less susceptible to AAA than those with C allele. Meanwhile, the rs1800896 (IL-10) locus had a positive association under the five statistical models, and individuals with A mutant gene at locus rs1800896 might have a higher susceptibility to AAA than those with G allele. Nevertheless, the rs1800947 (CRP), rs1205 (CRP), and rs1800629 (TNF) loci did not have positive correlation under the five statistical models, with no statistical significance. The results indicate that the gene polymorphisms at rs1800629, rs1800947, and rs1205 loci were not related to the AAA susceptibility.

**Conclusions:**

Gene polymorphisms in certain known inflammatory mediators related to AAA susceptibility might serve as potential predictive biomarkers for clinical applications. Moreover, SNP of inflammatory mediators relevant to abdominal aortic aneurysmal formation and progression need extensive investigations to confirm these results.

## 1. Introduction 

Abdominal aortic aneurysm (AAA) is a kind of vascular degenerative disease, which occurs mostly in middle-aged and elderly people. Apart from the factors such as smoking, age, hypertension and dyslipidemia, genetic background is also an important risk factor for AAA [1]. Typically, AAA degeneration of the abdominal aorta is a manifestation of a systemic process characterized by inflammation, apoptosis of smooth muscle cells, and destruction of elastin and collagen in the media and adventitia. AAA rupture is associated with a great mortality rate, and selective surgical repair is an effective and relatively safe intervention measure [2]. However, endovascular repair (EVAR) within the aneurysm has been currently evaluated as an alternative to open surgical repair [3]. At present, AAA shows highest incidence rate among aneurysms and exhibits a high incidence rate in cardiovascular diseases (CVDs) in the general population with increased patient suffering and the risks of rupture and death [4].

AAA has the characteristics of tissue structural destruction because of chronic inflammation with unknown causes [5]. Factors such as CRP, IL-6, IL-10 and TNF-α have important functions in host immunity and they also participate in modulating inflammation in AAA. On the other hand, CD4+T cells have been known to accumulate in the aneurysmal wall. Single nucleotide polymorphisms (SNPs) related to AAA have been identified within certain human genes that encode these crucial inflammatory elements [6]. The cytokines (including TNF-α, IL-1β, IL-2, IL-6, IL-8) levels in plasma, which show the correlation with the AAA pathogenic mechanism, have been identified as biomarkers for AAA onset. Most of such cytokines display remarkably high levels among AAA cases, while some of them are associated with the aneurysm size [7]. It has been reported previously that massive inflammatory infiltration occurs in the aneurysmal wall, which mostly consists of B cells, macrophages and T-cells. In addition to that, reactive oxygen species (ROS) contents are changed within AAA tissues compared to controls [8, 9]. Furthermore, some inflammatory factors show drastically high levels in the serum, which is related to aortic diameter in the elderly AAA male patients, as shown by ultrasound screening [10]. Nevertheless, it remains unclear whether the inflammation on AAA wall causes aneurysmal enlargement or is secondary to proteolysis.

C-reactive protein (CRP), the acute-phase protein, can also serve as the early marker for inflammatory disease or infection. Previous studies have investigated the associations between AAAs and circulating CRP levels with specific genetic polymorphisms [11]. For instance, the studies by Stephen *et al*. [12] showed that CRP modulated inflammation, and its expression increased among AAA cases, making CRP one of the prominent inflammatory factors aggravating AAA. Interleukin (ILs) are the lymphokines that interact with leukocytes or immune cells. Among them, IL-6, one of the pro-inflammatory factors, is suggested to show increased circulating levels in AAA cases, which may be associated with aorta diameter. Common genetic variations in the IL-6 gene promoter may affect the circulating IL-6 levels [13]. IL-10 can reduce pro-inflammatory cytokine production through macrophages, neutrophils and T cells. Its expression increases among AAA cases. Based on this IL-10 has turned out to be one of the susceptibility factors for AAA [14]. Tumor necrosis factor-alpha (TNF-α) can be released by T lymphocytes, macrophages and NK cells when activated. Notably, the TNF-α-308A allele *in vitro* displays increased activity relative to common G allele, which relates to higher TNF-α level [15].

At present, polymorphisms of several pro-inflammatory cytokines are identified as AAA-related. However, there is modest systemic and quantitative analysis on the relationship between polymorphism of inflammatory factors with AAA susceptibility. The present work carried out an integrative meta-analysis of the relevant published articles to investigate the relationship between the polymorphisms of inflammatory factors and AAA susceptibility. As there are inconsistent results from the published articles, the present meta-analysis was performed to clarify the relation of inflammatory factor SNPs with AAA susceptibility.

## 2. Materials and Methods

### 2.1 Searching Strategy

The present work was registered at PROSPERO (https://www.crd.york.ac.uk/PROSPERO; registration No., CRD42021259433) on 11th August, 2021. This work was carried out following the criteria of Meta-analysis Of Observational Studies in Epidemiology (MOOSE) [16] and guidelines of the Preferred Reporting Items for Systemic Reviews and Meta-Analysis (PRISMA) [17] (http://www.prisma-statement.org/).

Electronic databases PubMed and Web of Science (WOS) were systemically searched on May 5th, 2021. Two researchers (Z. Z. and H. W.) were independently assigned the task of reviewing the study related to the inflammatory mediators associated with human AAA. The review study was based on the Web of Science Core Collection database (via Web of Science [v.5.35]; 1926 to May, 2021) and MEDLINE database (via PubMed; 1966 to May, 2021).

The search strategies were summarized as follows, (“polymorphism” or “genetic polymorphism” or “single nucleotide polymorphism” or “SNP” or “genetic variants” or “gene mutation”) and (“abdominal aortic aneurysm” or “aortic aneurysm, abdominal” or “AAA”) and (“high sensitivity C-reactive protein” or “acute phase proteins” or “C Reactive Protein” or “CRP”) or (“interleukin-6” or “IL-6”) or (“interleukin-10” or “IL-10”) or (“TNF-alpha” or “TNF-α” or “tumor necrosis factor-alpha” or “tumor necrosis factor-α”) (**Supplementary File 1**). Only English language studies were retrieved. In addition, the reference lists from original studies and review papers were manually searched by the PubMed function to identify additional eligible articles.

### 2.2 Inclusion and Exclusion Criteria

Studies were selected according to the framework of PICOS (Population, Intervention/Exposure, Control, Outcomes, and Study design):

(1) Participants/population. Participants with the diagnosis of AAA by the local AAA screening project were enrolled.

(2) Intervention(s), exposure(s): (i) Serum samples were acquired in the whole blood from AAA cases and healthy subjects to measure the CRP, TNF-α, IL-6, and IL-10 levels. (ii) The alleles at diverse loci were analyzed for their relationship with clinical outcomes, so as to analyze the associations of genetic polymorphisms with clinical outcomes. There was at least one SNP with in inflammatory mediator genes related to AAA.

(3) Control groups. Participants from the screening project who were verified to have no AAA by ultrasound or medical imaging examination (e.g., CT) were selected in the control group.

(4) Outcome(s). Standard Mean Differences (SMDs) were used to calculate the correlations of circulating CRP, IL-6, IL-10, and TNF-α levels with AAA, and Odds Ratio (ORs) demonstrated the genetic relations with AAA. For all SNPs, their respective computations were completed by genotype frequencies and sample sizes in line with the recessive, dominant, as well as additive modes of inheritance.

(5) Study design: This study enrolled case-control or cohort studies that explored the relations of one or more SNPs of inflammatory mediator genes with the AAA risk.

Exclusion criteria:

(1) Studies that involved animal models;

(2) Studies that investigated participants suffering from concurrent connective tissue diseases (like Ehlers Danlos syndrome or Marfan’s syndrome);

(3) Duplicated studies;

(4) Reviews;

(5) Studies that mentioned non-inflammatory mediators.

### 2.3 Study Selection

Two reviewers (Z. Z. and H. Z.) selected the studies that were in line with the eligibility criteria for systematic review. Titles and abstracts of all the recruited articles were read by these two researchers, and differences in opinion between them were resolved by a third researcher (Y. H.). The Newcastle-Ottawa Scale (NOS) [18] was utilized to evaluate the quality of the enrolled studies. After NOS guideline modification, studies with the NOS score ≥6 stars were regarded as high-quality.

### 2.4 Data Extraction

Data were independently collected from the eligible studies by two researchers (Z. Z., and H. W.) by adopting the unified report form. Any disagreement between them was settled by a third author (Y. H). The collected data included the contents of circulating inflammatory mediators and their gene polymorphisms was also assessed. The study was grouped based on population characteristics, study design, author, year of publication, country, ethnicity, numbers of cases and controls, genotyping method, gender and genotype frequencies of each group. In addition, this study standardized SNP annotations by adopting the reference sequence (rs) numbers. dbSNP resource (http://www.ncbi.nlm.nih.gov/snp) was employed to assign the missing rs numbers by the NCBI server if available. Information on the polymorphisms of CRP, IL-6, IL-10, and TNF-α was collected in the dbSNP_NCBI, PubMed, public version of Human Gene Mutation Database (HGMD), and Functional Single Nucleotide Polymorphism (F-SNP) database. The spreadsheet (Microsoft Excel 2010; Microsoft, Redmond, WA, USA) was utilized to record the data collection means.

### 2.5 Heterogeneity Evaluation

This study applied *$I^{2}$* test in evaluating the possible heterogeneity; with *$I^{2}$* = 25%, 25%–50%, >50%, indicating low, moderate and severe heterogeneity, respectively [19]. Additionally, subgroup analysis was conducted to explore the potential source of heterogeneity when there was obvious heterogeneity across diverse studies. The countries involved were European countries such as the United Kingdom and Sweden; Australia and other Oceanian countries; China and other Asian countries.

### 2.6 Statistical Analysis

SMDs were used to calculate the correlations between circulating CRP, IL-6, IL-10, and TNF-a levels and the AAA susceptibility. *p*-values were calculated by Mantel-Haenszel statistical approach under the allele, homozygous, heterozygous, dominant and recessive models. Unadjusted ORs were calculated based on original data on genotype frequencies, and were pooled for meta-analysis by using the random effects model. Allele and genotype comparisons study separately assessed the risks of variant vs. wild-type (WT) alleles, heterozygote vs. WT homozygotes, and variant vs. WT homozygotes. Subsequently, our study individually evaluated the risks of recessive and dominant effects for variant allele (heterozygote + variant homozygote vs. WT homozygote and variant homozygote vs. WT homozygote + heterozygote). Due to the small number of rare homozygotes, overestimated CIs were obtained by adopting the recessive model of analysis compared with the dominant model [20]. *p *< 0.05 indicated that the polymorphisms of inflammatory factors such as CRP and IL-6 were significantly related to the risk of AAA. In the instance when there was obvious heterogeneity, we conducted sensitivity analysis for analyzing factors affecting enrolled study homogeneity. Egger’s test was also performed to assess the possible publication bias by the 95% CI. Stata 16.0MP version (version 16.0; College Station, TX, USA) was adopted for statistical analysis.

## 3. Results

### 3.1 General Information and Quality Evaluation of the Included Studies

**Fig. 1.**
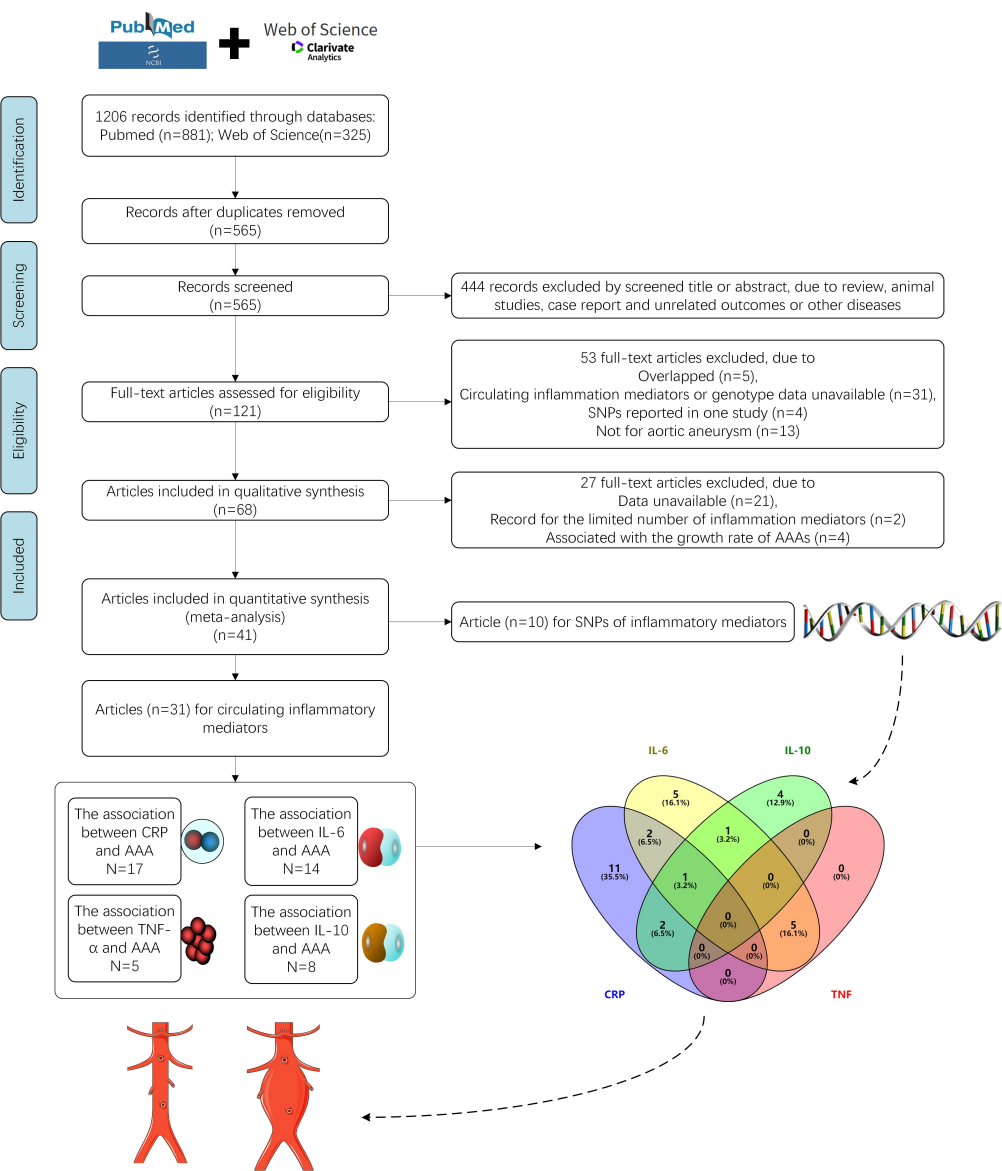
**Flow chart demonstrating study inclusion and exclusion criteria**.

The study inclusion and exclusion flow chart of this systemic review and meta-analysis is presented in Fig. 1, which is in line with the PRISMA group. According to the search strategies, 121 relevant documents were preliminarily obtained after removing 444 duplicates. Post screening, 88 articles met the standard, and a total of 41 citations were determined. After full-text screening, 20 studies were found that reported the associations between CRP level/SNPs and AAA susceptibility, 17 mentioned the relations of IL-6 level/SNPs with AAA susceptibility, 7 reported the relationship between TNF-a level/SNPs and AAA susceptibility, and 10 mentioned the associations of IL-10 level/SNPs and AAA susceptibility.

Overall, 6 SNPs in four genes representing inflammatory mediators were examined (Table 1, Ref. [11, 12, 13, 14, 15, 21, 22, 23, 24, 25]). Table 1 summarizes the previously reported associations between SNPs in CRP, IL-6, IL-10, and TNF-α genes with the AAA susceptibility. Out of these, six SNPs in four genes (rs3091244, CRP; rs1800947, CRP; rs1205, CRP; rs1800795, IL-6; rs1800896. IL-10; rs1800629, TNF) were analyzed in our meta-analysis, including four that were evaluated in four articles, whereas 2 SNPs were mentioned in one article. Most of these SNPs were analyzed by whole coding region sequencing. Table 2 (Ref. [10, 12, 21, 23, 26, 27, 28, 29, 30, 31, 32, 33, 34, 35, 36, 37, 38, 39, 40, 41, 42, 43, 44, 45, 46, 47, 48, 49, 50, 51, 52]) summarizes demographic and clinical data of subjects evaluating levels of inflammation mediators in patients with AAA and control subjects.

**Table 1.** **Demographic and clinical data of subjects enrolled in studies evaluating SNPs of inflammation mediators in patients with AAA and control subjects**.

Author	Year	Group	Number	Study	Country	Inflammatory	Detection method	Gene	Genotype	Genotyping method	Age (years)	Smoking (n)	Male/Females (n)	Hypertension (n)	BMI (kg⁄$m^{2}$)	Diabetes (n)	Dyslipidemia
Badger *et al*. [12]	2009	Case	248	Case-control	UK	CRP		rs3091244	TT/AA/TA = 33; CT/CA = 108; CC = 107			174					
		Control	400						TT/AA/TA = 46; CT/CA = 182; CC = 172			228					
Smallwood *et al*. [13]	2008	Case	677	Case-control	AUS	IL-6		rs1800795	GG = 222; GC = 300; CC = 104	TaqMan	73.3	579			27.2	67	
		Control	656						GG = 224; GC = 302; CC = 124		72.3	420			26.6	38	
Bown *et al*. [23]	2007	Case	389	Case-control	UK	IL-10	ELISA	rs1800896	AA = 104; GA = 201; GG = 84			460	355/34	282		31	
		Control	404						AA = 81; GA = 205; GG = 118			323	395/9	179		37	
Duellman *et al*. [24]	2014	Case	141	Case-control	USA	IL-10		rs1800896	AA = 42; GA = 60; GG = 39								
		Control	168						AA = 48; GA = 77; GG = 43			82	74/94	94	29.3 ± 0.4		83
Wang *et al*. [25]	2015	Case	425	Case-control	China	IL-10		rs1800896	AA = 64; GA = 161; GG = 156	PCR-RFLP		273	315/66	256			
		Control	381						AA = 46; GA = 151; GG = 184			209	315/66	166			
Saratzis *et al*. [11]	2013	Greece Case	351	Case-control	Greece	CRP	Nephelometry	rs3091244	TT/AA/TA = 70; CT/CA = 165; CC = 116		69 ± 8	257	322/29	271		77	
		Greece Control	391						TT/AA/TA = 35; CT/CA = 129; CC = 227		73 ± 5	311	327/64	300		78	
		UK Case	371		UK	CRP			TT/AA/TA = 82; CT/CA = 193; CC = 96		72 ± 7	326	347/24	187		58	
		UK Control	362						TT/AA/TA = 54; CT/CA = 167; CC = 141		71 ± 7	302	345/17	178		52	
Shangwei *et al*. [22]	2017	Case	155	Case-control	China	CRP		rs1800947	GG = 0; GC = 8; CC = 143		69.2 ± 9.9	132	138/17	108			118
									GG = 0; GC = 16; CC = 286		69.5 ± 9.9	167	276/34	143			138
		Control	310					rs1205	TT+TC = 127; CC = 23		69.2 ± 9.9	132	138/17	108			118
									TT+TC = 249; CC = 53		69.5 ± 9.9	167	276/34	143			138
Jabłońska *et al*. [15]	2020	Case	104	Case-control	Austria	IL-6	ELISA	rs1800795	GG = 24; GC = 52; CC = 28	PCR-RFLP	70.5 ± 7.0	61			27.54 ± 4.29		
									GG = 43; GC = 46; CC = 23		69.7 ± 9.6	11			27.41 ± 4.20		
		Control	112			TNF-α		rs1800629	AA = 12; GA = 47; GG = 45		70.5 ± 7.0	61			27.54 ± 4.29		
									AA = 10; GA = 32; GG = 70		69.7 ± 9.6	11			27.41 ± 4.20		
Bown *et al*. [14]	2003	Case	100	Case-control	UK	IL-6		rs1800795	GG = 33; GC = 48; CC = 19	PCR-RFLP, SSP		84		55		6	20
									GG = 28; GC = 57; CC = 15			73		36		15	14
						TNF-α		rs1800629	AA = 6; GA = 30; GG = 64			84		55		6	20
		Control	100						AA = 5; GA = 32; GG = 63			73		36		15	14
						IL-10		rs1800896	AA = 34; GA = 49; GG = 17			84		55		6	20
									AA = 23; GA = 48; GG = 29			73		36		15	14
Qin *et al*. [21]	2018	Case	155	Case-control	China	CRP		rs1800947	GG = 0; GC = 8; CC = 143	Sequenom’s Mass-ARRAY	69.2 ± 10.0	132	138/17	108	24.4 ± 3.4		76
									GG = 1; GC = 8; CC = 144		69.6 ± 10.9	69	138/17	64	25.3 ± 3.2		34
		Community Control	155						GG = 0; GC = 8; CC = 143		69.2 ± 10.0	132	138/17	108	24.4 ± 3.4		76
									GG = 1; GC = 8; CC = 142		69.5 ± 9.0	98	138/17	79	24.2 ± 3.5		27
								rs1205	TT = 51; TC = 76; CC = 23		69.2 ± 10.0	132	138/17	108	24.4 ± 3.4		76
		Hospital Control	155						TT = 48; TC = 73; CC = 31		69.6 ± 10.9	69	138/17	64	25.3 ± 3.2		34
									TT = 51; TC = 76; CC = 23		69.2 ± 10.0	132	138/17	108	24.4 ± 3.4		76
									TT = 48; TC = 80; CC = 22		69.5 ± 9.0	98	138/17	79	24.2 ± 3.5		27

**Table 2.** **Demographic and clinical data of subjects enrolled in studies evaluating levels of inflammation mediators in patients with AAA and control subjects**.

Author	Year	Group	Number	Study	Country	Inflammatory	Detection method	Age (years)	Smoking (n)	Male/Females (n)	Hypertension (n)	BMI (kg⁄$m^{2}$)	Diabetes (n)	Dyslipidemia (n)
Palazzuoli *et al*. [35]	2008	Case	98	Case-control	Italy	CRP		74 ± 8	59	76/22		29 ± 3.8		
		Control	82					74 ± 8	31	50/32		28 ± 3.6		
Cersit *et al*. [26]	2021	Case	150	Case-control	Turkey	CRP		66.8 ± 10.6	38	117/33	58	27.1 ± 3.2	57	61
		Control	100					64.7 ± 8.6	23	75/25	30	25.5 ± 2.6	32	36
Dawson *et al*. [52]	2007	Case	25	Case-control	UK	CRP		73		27/0			4	
		Control	12					50		3/12			0	
Golledge *et al*. [28]	2007	Case	318	Case-control	AUS	CRP	ELISA							
		Control	634											
Wanhainen *et al*. [40]	2005	Case	35	Case-control	Sweden	CRP							3	
		Control	140										17	
Parry *et al*. [37]	2010	Case	75	Case-control	UK	CRP and IL-6	ELISA	72				26.95	13	
		Control	90					72				27.32	2	
Golledge *et al*. [27]	2010	Case	312	Case-control	AUS	CRP		71.8	268		158		21	150
		Control	1046					72.8	650		429		78	366
Pan *et al*. [36]	2011	Case	45	Case-control	China	CRP		76	31	39/6	21	23.7 ± 2.9	7	
		Control	49					74	24	41/8	14	24.8 ± 3.4	2	
Lindqvist *et al*. [34]	2012	Nonruptured AAA Case	78	Case-control	Sweden	CRP and IL-6	ELISA	71	34	62/16				
		Control	36					72	15	30/6				
		Ruptured AAA Case	41					73	17	33/8				
Ramos-Mozo *et al*. [38]	2012	Case	30	Case-control	Spain	CRP	ELISA	69 ± 5	17	30/0	15		3	9
		Control	30					67 ± 5	12	29/1	19		5	16
Kadoglou *et al*. [32]	2012	Case	108	Case-control	Greece	CRP, IL-6, and IL-10		72 ± 4	41			28.98 ± 4.23	21	
		Control	42					69 ± 8	6			29.36 ± 5.79	12	
Hellenthal *et al*. [30]	2012	Small AAA Case	59	Case-control	Netherlands	CRP	ELISA	70.1 ± 7.4	55	45/14	40			4
		Control	69					71.6 ± 5.4	30	59/10	24			24
		Medium AAA Case	64					71.7 ± 7.9	58	55/9	40			8
		Control	69					71.6 ± 5.4	30	59/10	24			24
		Large AAA Case	95					72.7 ± 7.5	90	89/6	52			7
		Control	69					71.6 ± 5.4	30	59/10	24			24
Treska *et al*. [46]	2002	Case	32	Case-control	AUS	IL-6 and TNF-α								
		Control	14											
Qin *et al*. [21]	2013	Case	31	Case-control	China	CRP	ELISA	63.45 ± 12.83	19	25/6	22	26.26 ± 9.41		
		Control	32					58.88 ± 7.12	7	15/17	1	24.55 ± 3.42		
Jones *et al*. [31]	2016	Case	442	Case-control	New Zealand	CRP and IL-10		75.0 ± 7.9		334/108	262		50	
		Control	970					68.5 ± 7.6		741/229	307		66	
Sohrabi *et al*. [39]	2014	Case	86	Case-control	UK	CRP		73		67/19	48	27	11	
		Control	158					71		114/44	73	27	22	
Wong *et al*. [41]	2013	Case	311	Case-control	AUS	CRP		77.7	292		2971	27	67	255
		Control	3922					76.5	2549		247	26.5	596	2773
Golledge *et al*. [29]	2007	Case	233	Case-control	AUS	CRP	ELISA	76.0 ± 5.9	188	233/0	113		18	149
		Control	233					74.6 ± 7.4	145	233/0	81		12	122
Wallinder *et al*. [49]	2009	Small AAA Case	38	Case-control	Sweden	IL-6 and IL-10		70	16	27/11				
		Control	41					72	18	33/8				
		EAAA Case	40					71	19	35/5				
		Control	41					72	18	33/8				
Fowkes *et al*. [44]	2006	Case	89	Case-control	UK	IL-6	ELISA	73.5 ± 0.5	79	64/25		25.0 ± 0.4		
		Control	98					73.5 ± 0.5	67	70/28		26.3 ± 0.4		
Juvonen *et al*. [45]	1997	Case	50	Case-control	Finland	IL-6	Radioimmunoassay RIA			40/10				
		Control	38							17/21				
Ahnström *et al*. [42]	2010	Case	343	Case-control	Sweden	IL-6		74 ± 8	119	271/72		25.4 ± 4.05	41	
		Control	214					68 ± 2	26	99/115		27.1 ± 4.33	12	
Lindberg *et al*. [10]	2016	Case	116	Case-control	Sweden	IL-6 and TNF-α	ELISA							
		Control	239											
Buffa *et al*. [43]	2019	Case	60	Case-control	Italy	IL-6	ELISA	70	42	40/20	38	25.9	10	12
		Control	80					72	5	35/45	2	25.8	0	2
Liao *et al*. [33]	2015	Small AAA Case	385	Case-control	Denmark	CRP and IL-10	ELISA							
		Control	200											
		Large AAA Case	91											
		Control	200											
Aria *et al*. [50]	2018	Case	5	Case-control	Iran	IL-10								
		Control	5											
Windsor *et al*. [51]	2017	Case	20	Case-control	AUS	IL-10	ELISA	74	13		14	27	1	16
		Control	20					71	11		5	26	0	6
Treska *et al*. [47]	2007	Ruptured AAA Case	54	Case-control	Czech	IL-6 and TNF-α								
		Control	15											
		Asymptomatic ruptured AAA Case	41											
Treska *et al*. [48]	2011	Case	345	Case-control	Unknown	IL-6 and TNF-α								
		Control	30											
Badger *et al*. [12]	2009	Case	248	Case-control	UK	CRP			174					
		Control	400						228					
Bown *et al*. [23]	2007	Case	389	Case-control	UK	IL-10	ELISA		460	355/34	282		31	
		Control	404						323	395/9	179		37	

Table 1 displays the basic study characteristics. All the recruited articles were observational studies that were published from year 2000 to 2019 involving 9007 AAAs patients and 14,315 normal control individuals. Among them, 40 articles reported the circulating inflammatory mediators enrolled in this meta-analysis (n = 20 for CRP, n = 17 for IL-6, n = 8 for IL-10, and n = 9 for TNF-α), whereas 10 SNPs were mentioned from inflammatory mediators enrolled in this meta-analysis. In each article, included subjects were from Asia, Europe, North America and Australasia that were enrolled. Typically, the existence of AAA was confirmed mostly by ultrasonography [53], but it was verified by CT angiography in one study. Table 1 displays the characteristics and method wise quality of all the enrolled articles.

### 3.2 Circulating CRP Level and CRP (rs3091244, rs1800947, rs1205) Polymorphisms

#### 3.2.1 CRP Level and Subgroup Analysis

**Fig. 2.**
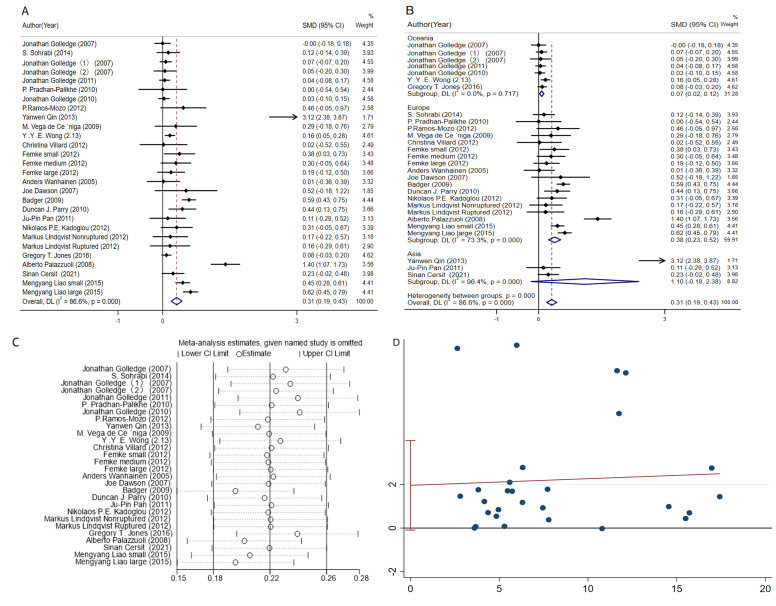
**The forest plot illustrating the SMD and 95% CI for the association between circulating CRP levels and abdominal aortic aneurysm**. (A) Meta-analysis of plasma CRP levels. (B) Subgroup analysis of plasma CRP levels. (C) Sensitivity analysis of plasma CRP levels. (D) Egger test of plasma CRP levels.

Initially, the circulating CRP level was used as a risk factor to explore whether the plasma CRP level affected the occurrence of AAAs. According to our results, the SMD was 0.30 mg/L (95% CI: 0.17–0.43, *p <* 0.001, Fig. 2A) when using the random effects model, which indicated that CRP level affected the risk of AAA. In other words, the plasma levels of CRP increased among AAA cases [12, 26, 27, 28, 29, 30, 31, 32, 33, 34, 35, 36, 37, 38, 39, 40, 41]. Subgroup analysis was also carried out based on the continents in terms of where the study objects were located. It was observed that the CRP level affected the European and Oceanian populations in particular (Fig. 2B).

Sensitivity analysis (Fig. 2C) was also carried out. It was found that each of the eliminated studies slightly affected the pooled results, with no obvious change in the impact of every single study, thus substantiating the result of our analysis.. Egger’s test was performed to identify the potential source of publication bias. As shown in Fig. 2D, *p* = 0.793 was obtained, indicating no significant evidence of publication bias.

#### 3.2.2 CRP (rs3091244, rs1800947, rs1205) Polymorphisms 

**Fig. 3.**
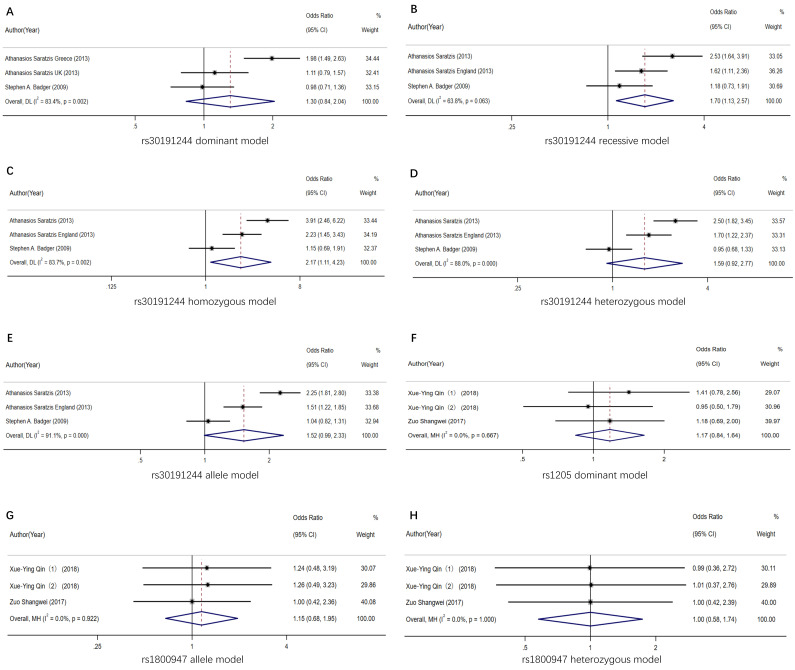
**The forest plot demonstrating the OR and 95% CI association between CRP rs3091244, rs1205, rs1800947 and abdominal aortic aneurysm**. (A) rs3091244 dominant gene model. (B) rs3091244 recessive gene model. (C) rs3091244 homozygous model. (D) rs3091244 heterozygous model. (E) rs3091244 allele model. (F) rs1205 dominant gene model. (G) rs1800947 allele model. (H) rs1800947 heterozygous model.

It was found that the CRP allele rs3091244 (minor allele frequency = 36.8%) was significantly associated with AAA and the recessive models of inheritance (A/A+T/T +A/T vs A/C+T/C+C/C, *$I^{2}$* = 63.8%, OR = 1.70, 95% CI: 1.13–2.57; *p* = 0.011, Fig. 3A). In contrast, CRP allele rs3091244 was not significantly associated with AAA and the dominant model of inheritance (OR = 1.30, 95% CI: 0.84–2.04, *$I^{2}$* = 83.4%, *p* = 0.242) (Fig. 3B). There was statistical significance of ORs obtained for additive model (A/A+T/T+A/T vs C/C, OR = 2.17, 95% CI: 1.11–4.23, $I^{2}$ = 83.7%, *p* = 0.023) for the homozygous model (Fig. 3C), but not for the heterozygous (OR = 1.59, 95% CI: 0.92–2.77, *p* = 0.097; Fig. 3D) or allele model (Fig. 3E) [11, 12]. In addition, subgroup analysis stratified by the country of the statistical population was also conducted as shown by the forest plot in **Supplementary File 2**. A and T mutant genes of rs3091244 clearly show a higher susceptibility tendency to AAA than individuals with C allele. The Greek population study was even more convincing. According to Egger’s test for publication bias, there was no obvious evidence of publication bias.

*p *> 0.05 was obtained under the rs1205 dominant allele model, which indicated no statistical significance. The forest plot under the dominant gene model of inheritance (T/T+T/C vs C/C, OR = 1.17, 95% CI: 0.84–1.64, $I^{2}$ = 0%, *p *= 0.347) is presented in Fig. 3F. According to the results obtained from the forest plot, different genotypes at this locus did not affect the susceptibility to AAAs [21, 22].

Difference in the rs1800947 locus showed no significant difference between the allele and the heterozygous models (*p *= 1.000). The forest plot is exhibited in Fig. 3G,H. Under the allele model (G vs C), the susceptibility to AAAs was not significantly related to the dominant model of inheritance (OR = 1.15, 95% CI: 0.68–1.95, *$I^{2}$* = 0.0%, *p *= 0.604) (Fig. 3G). Statistical significance was detected in ORs obtained for the additive model (G/C vs C/C, OR = 1.00, 95% CI: 0.58–1.74, $I^{2}$ = 83.7%, *p *= 1.000) for the homozygous model (Fig. 3H). It was concluded that there was no difference in AAA susceptibility between individuals with rs1800947G mutant gene and those with C allele [21, 22].

### 3.3 Circulating IL-6 Level and IL-6 (rs1800795) Polymorphisms

#### 3.3.1 Circulating IL-6 Level

**Fig. 4.**
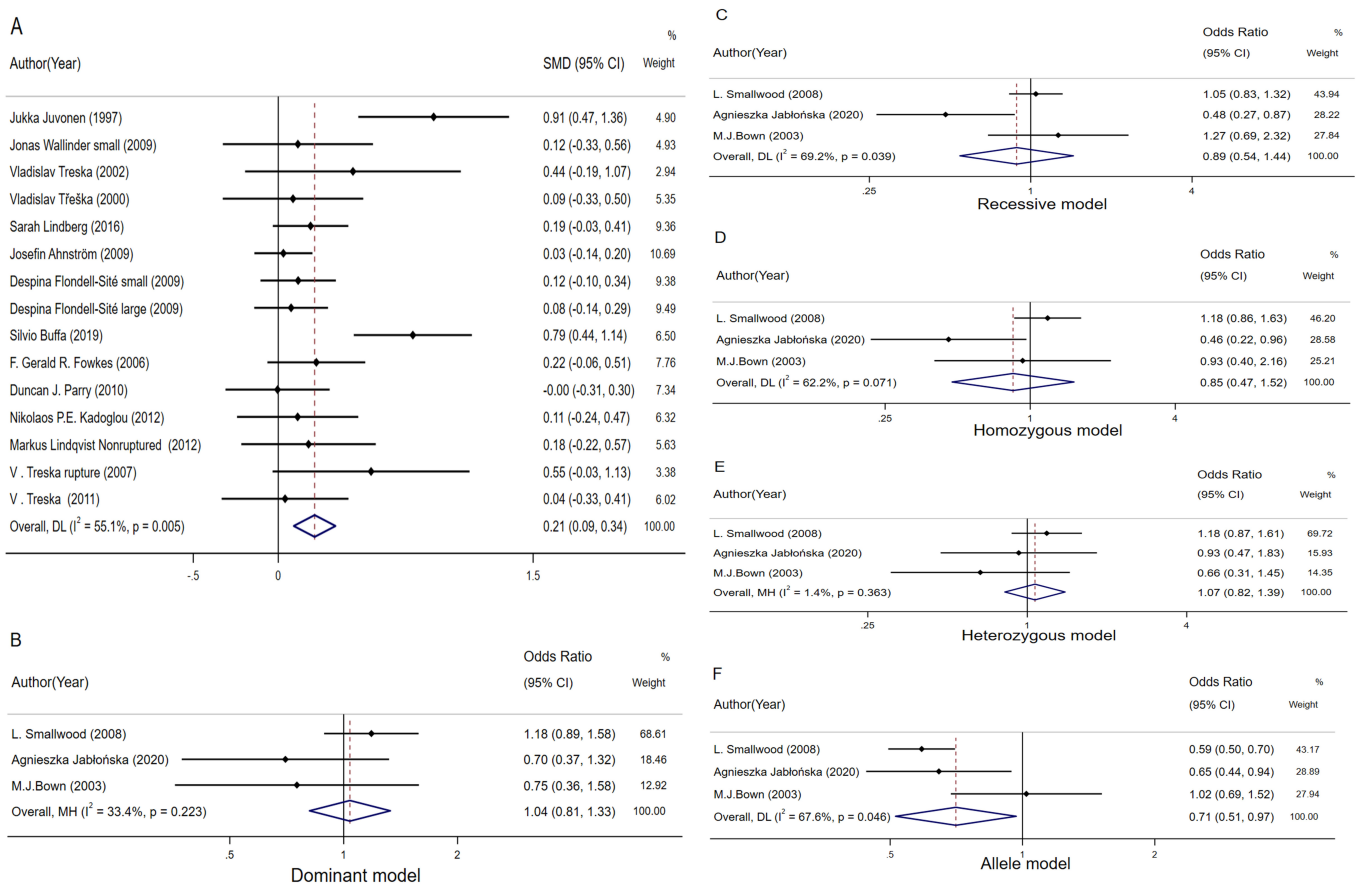
**The forest plot comparing the SMD and 95% CI association between circulating interleukin-6 levels and abdominal aortic aneurysm, and the OR and 95% CI association between rs1800795 and abdominal aortic aneurysm**. (A) Meta-analysis of plasma IL-6 levels. (B) rs1800795 dominant gene model. (C) rs1800795 recessive gene model. (D) rs1800795 homozygous model. (E) rs1800795 heterozygous model. (F) rs1800795 allele gene model.

A total of 17 relevant studies were included in the IL-6 group. Interestingly, the circulating IL-6 levels of AAA patients were dramatically elevated compared to the control group (SMD = 0.34, 95% CI: 0.20–0.49, $I^{2}$ = 74.2%, *p *< 0.001, Fig. 4A). This indicated that the IL-6 level had a risk effect on the occurrence of AAAs. According to subgroup analysis stratified by the continents, where the study objects were located, IL-6 levels affected the Asian, European and Oceanian populations. The forest plot is displayed in **Supplementary File 3**. It should be noted that additional data is needed to confirm the corresponding results for the Asian population [7, 10, 32, 34, 37, 42, 43, 44, 45, 46, 47, 48, 49].

Sensitivity analysis was also conducted in order to explore the potential heterogeneity source. As a result, the inclusion of each study had little effect on the pooled results. The Egger’s test was also performed, as shown in **Supplementary File 3**, and the *p*-value was 0.469, revealing low publication bias and reliable analysis results.

#### 3.3.2 IL-6 (rs1800795) Polymorphisms

This study analyzed the relationship between the IL-6 locus rs1800795 and the risk of AAA. The dominant gene model (G/G+G/C vs C/C, OR = 1.04, 95% CI: 0.81–1.33, $I^{2}$ = 33.4%; *p* = 0.763, Fig. 4B) and the recessive gene model (G/ G vs C/C+G/C, OR = 0.89 (95% CI: 0.54–1.44, $I^{2}$ = 69.2%, *p* = 0.626, Fig. 4C), indicated that IL-6 SNP was not related to AAA susceptibility. For GG, there was no difference in the susceptibility to AAA between the populations of CC and GC genotypes [13, 14, 15]. Meanwhile, the risk of AAA was compared between the homozygous model (G/G vs. C/C, OR = 0.85 (95% CI: 0.47–1.52, $I^{2}$ = 62.2%), *p* = 0.581, Fig. 4D) and the heterozygous model (G/C vs C/C, OR = 1.07, 95% CI: 0.82–1.39, $I^{2}$ = 1.4%, *p* = 0.617, Fig. 4E). The result confirmed the dominant and the recessive model, however, a decreased AAA susceptibility was observed in allele carriers with IL-6/rs1800795 genotype (G vs C, OR = 0.71; 95% CI: 0.51–0.97; $I^{2}$ = 67.6%, *p* = 0.011, Fig. 4F).

It was assessed that individuals carrying the G mutant gene of rs1800795 might be less susceptible to AAA than those with C allele, with greater impact on the Australian and Austrian populations. The Egger’s test was also conducted to test the publication bias, as shown in **Supplementary File 3**. Small publication bias was detected, indicating that the results of this meta-analysis had a certain degree of credibility.

### 3.4 Circulating IL-10 Level and IL-10 (rs1800896) Polymorphisms

#### 3.4.1 Circulating IL-10 Level

**Fig. 5.**
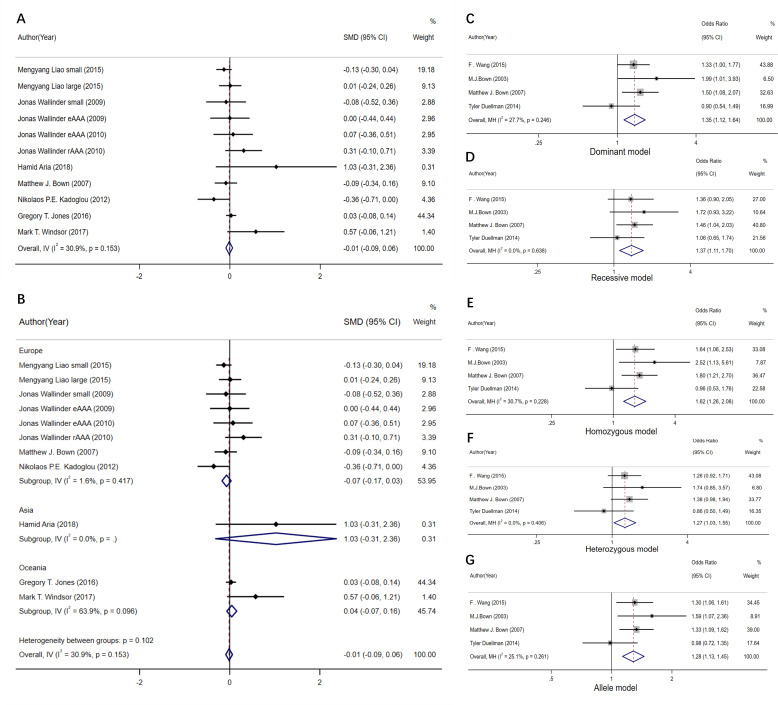
**The forest plot comparing the SMD and 95% CI association between plasma interleukin-10 levels and abdominal aortic aneurysm, and the OR and 95% CI association between rs1800896 and abdominal aortic aneurysm**. (A) Meta-analysis of plasma IL-10 levels. (B) Subgroup analysis of plasma IL-10 levels. (C) rs1800896 dominant gene model. (D) rs1800896 recessive gene model. (E) rs1800896 homozygous model. (F) rs1800896 heterozygous model. (G) rs1800896 allele model.

A total of 8 studies were included, and no statistically significant increase or decrease in circulating IL-10 levels were observed between AAA patients in comparison with controls (SMD = –0.01, 95% CI: –0.09–0.06, $I^{2}$ = 30.9%,* p *= 0.710, Fig. 5A). Subgroup analysis was also conducted, and the results indicated that the IL-10 level did not affect the occurrence of AAA, and this conclusion was also applicable to the European, Asian and Oceanian populations (Fig. 5B) [23, 31, 32, 33, 49, 50, 54, 51]. This study also carried out sensitivity analysis, which revealed no significant change in the effect of size of each study, thereby proving the results of this meta-analysis. The Egger’s test for publication bias was performed on this set of data, as shown in **Supplementary File 4**.

#### 3.4.2 IL-10 (rs1800896) Polymorphisms

At the same time, sensitivity analysis was performed on the IL-10 locus rs1800896. Compared to healthy subjects, the dominant gene model of IL-10/rs1800896 SNP (A/A+A/G vs G/G) was more prevalent among AAA patients (OR = 1.35, 95% CI: 1.12–1.64, $I^{2}$ = 27.7%, *p* = 0.002, Fig. 5C). With regard to the recessive gene model (A/A vs G/G+A/G), *IL-10 (rs1800896)* SNP among AAA patients was more effective than that in healthy patients. In addition to that, the occurrence of this SNP was more frequent among the subjects (OR = 1.37, 95% CI: 1.11–1.70, $I^{2}$ = 0%, *p* = 0.004, Fig. 5D), indicating that patients with AA and AG genotypes were more likely to develop AAA than those with GG genotype. However, patients with AA genotype were more susceptible to AAA than those with AG and GG genotypes [14, 23, 24, 25].

AAA cases showed an increased heterozygous genotype rate for *IL-10 (rs1800896)* SNP (A/A vs G/G) compared to normal controls (OR = 1.62, 95% CI: 1.26–2.08, $I^{2}$ = 30.7%, *p *< 0.001; Fig. 5E). Population with AA genotype had a higher susceptibility to AAA than that with GG genotype. However, in the heterozygous model (A/G vs G/G), our results showed that the risk of AAA was different in the populations carrying the A and G alleles (OR = 1.27, 95% CI: 1.03–1.55, $I^{2}$ = 0%, *p* = 0.022; Fig. 5F), and the population with AG genotype had a higher rate than that of GG genotype. The allele genotype of the *IL-10 (rs1800896)* SNP (A vs G) showed a higher frequency among AAA cases compared to normal controls (OR = 1.28, 95% CI: 1.13–1.45, $I^{2}$ = 25.1%, *p *< 0.001; Fig. 5G). The above results suggest that the risk of AAA increased in populations carrying the A and G alleles, and that the A allele at the rs1800896 locus of IL-10 was more susceptible to AAA than the G allele.

Based on the above results, it was inferred that the A mutant gene might have a higher susceptibility to AAA than the G allele, which also had a greater impact on the Chinese and British populations. Therefore, sensitivity analysis was further extended, and Egger’s test for publication bias was performed on this set of data under the allelic model. The analysis indicated a certain degree of credibility in our results, as shown in** Supplementary File 4**.

### 3.5 Circulating TNF-α Level and TNF (rs1800629) Polymorphisms

#### 3.5.1 Circulating TNF-α Level 

**Fig. 6.**
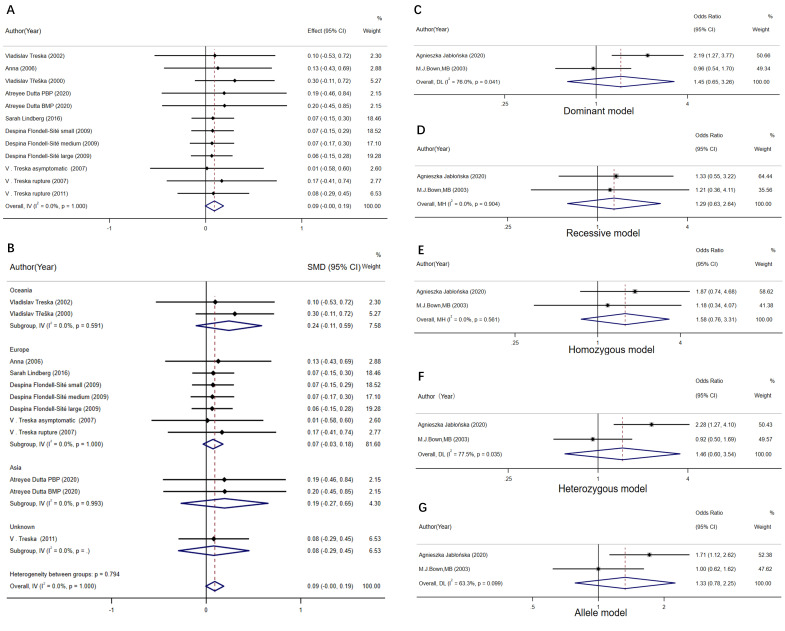
**The forest plot comparing the SMD and 95% CI association between plasma TNF-α levels and abdominal aortic aneurysm, and the OR and 95% CI association between rs1800629 and abdominal aortic aneurysm**. (A) Meta-analysis of plasma TNF-α levels. (B) Subgroup analysis of plasma TNF-α levels. (C) rs1800629 dominant gene model. (D) rs1800629 recessive gene model. (E) rs1800629 homozygous model. (F) rs1800629 heterozygous model. (G) rs1800629 allele model.

8 studies were included to investigate circulating TNF-α Level which showed no statistically significant increase/decrease in the circulating IL-10 level between the AAA group compared to the control group SMD = 0.09, 95% CI: 0.00–0.19, *p* = 0.062; Fig. 6A). Subgroup analysis was also conducted based on the continents where the populations was located, as shown in Fig. 6B. According to subgroup analysis, such a result was applicable to both European and Asian populations [7, 10, 46, 47, 48]. Sensitivity analysis was also carried out, indicating that there was no significant change in the effect of size of each study, proving the reliability of results of this meta-analysis. Moreover, Egger’s test for publication bias was performed on this set of data, as shown in **Supplementary File 5**. The *p*-value was 0.548, indicating that there was no significant publication bias.

#### 3.5.2 TNF (rs1800629) Polymorphisms 

This study also analyzed the susceptibility of TNF-α locus rs1800629 to AAA, as shown in Fig. 6C,D. There was no statistical significance based on the dominant gene model (A/A+A/G vs G/G, OR = 1.45, 95% CI: 0.65–3.26, $I^{2}$ = 76.0%, *p* = 0.363; Fig. 6C) or the recessive gene model (A/A vs A/G+G/G), OR = 1.29, 95% CI: 0.63–2.64, $I^{2}$ = 0%, *p* = 0.488, Fig. 6D). The final results revealed no difference in AAA susceptibility among populations with AA, AG and GG genotypes [14, 15]. Under the homozygous (A/A vs G/G), heterozygous (A/G vs G/G), and allele (A vs G) models, no significant difference in AAA risk was observed between AA genotype and GG genotype, or between AG genotype and GG genotype (OR = 1.58, 95% CI: 0.76–3.31, *p* = 0.2222; Fig. 6E; OR = 1.46, 95% CI: 0.60-3.54, *p* = 0.406; Fig. 6F; and OR = 1.33, 95% CI: 0.78–2.25, *p* = 0.294; Fig. 6G). Due to the small number of studies included, the results of this analysis might not be precise, so it was decided not to test the publication bias by Egger’s and Begg’s tests.

## 4. Discussion

Several articles identifying the AAA-related genetic risk factors have been published [55, 56]. Numerous previous studies have reported that the levels of various inflammatory factors or cytokines (ILs, TNF, and NO) and their polymorphisms are closely associated with AAA onset. However, the relationships between inflammatory factor gene SNPs and AAA susceptibility remain unknown because inconsistent results have been obtained from studies having small sample sizes. Currently, it is still not conclusive regarding the significance of results in each published study. This work aimed to explore the associations of inflammatory mediator levels and their gene polymorphisms with the susceptibility to AAAs, and summarize the susceptibility factors of AAAs. Firstly, this study examined the levels of four inflammatory factors in human AAA. In this study, healthy subjects were enrolled in the control group, whereas AAA patients were enrolled in the experimental group to detect their circulating levels of CRP, IL-6, IL-10, and TNF-α. Secondly, a meta-analysis was conducted on the SNP loci of various inflammatory factors and the statistics of their genotype distribution compared to healthy volunteers enrolled in the control group, and AAA patients in the experimental group.

### 4.1 Levels of Inflammatory Mediators and the Underlying Mechanisms

In this study we initially analyzed the levels of four continuous variables (CRP, IL-6, IL-10, and TNF-α) of inflammatory mediators. Circulating CRP contents among AAA cases remarkably increased compared to normal controls. Significantly increased IL-6 levels were measured among AAA cases compared to normal controls. In contrast, IL-10 and TNF-α levels were not associated with the risk of AAAs. The expression of acute-phase reactants is up-regulated during chronic or acute inflammation. Inflammation has been suggested as the important factor causing elastin decomposition and AAA progression [57], and the increased baseline CRP level can be detected among AAA cases. The present meta-analysis suggests that CRP levels are significantly higher among AAA cases (*p *< 0.001), while it is previously reported in a meta-analysis that CRP was related to the risk of AAAs [58]. CRP is mostly generated via hepatocytes, as well as aneurysmal tissue [59]. This hypothesis is supported by some of the results, because advanced aneurysms exhibit the greatest CRP levels [12]. Notably, CRP content is associated with the prevalence of CVDs. In addition, CRP has been suggested to modulate fibrinolysis, alter inflammatory molecule levels, and regulate processes involved in atheromatosis and coronary heart disease (CHD) formation [60, 61]. Subgroup analysis stratified by location was conducted, and it was found that CRP level had an impact on the AAA susceptibility in the European and Oceanian populations, but not in the Asian population.

This meta-analysis also indicated that the IL-6 level had a significant effect on the occurrence of AAA. It was also found that the IL-6 levels were associated with AAA susceptibility in the Asian, European and Oceanian populations by subgroup analysis. IL-6 is the pro-inflammatory factor that plays a critical role in triggering systemic inflammatory response [62]. As revealed by our meta-analysis results, the circulating IL-6 levels were dramatically elevated among AAA cases (*p* = 0.030); likewise, IL-6 levels were elevated among AAA patients, while IL-10 levels were not significantly changed, which was supported by results of IL-6 obtained from aortic tissues [63]. IL-10 is a strong anti-inflammatory factor, and the imbalance between IL-6 and IL-10 may interpret the inflammatory heterogeneities between AAA patients and normal subjects [64]. Recently, a relevant study also reported that the circulating IL-10 and TNF-α levels are not related to AAA susceptibility [58].

### 4.2 SNPs of Inflammatory Mediators and the Underlying Mechanisms

In this study, six SNP loci (rs3091244, rs1800947, rs1205 for CRP; rs1800795 for IL-6; rs1800896 for IL-10; and rs1800629 for TNF) were analyzed.

#### 4.2.1 SNPs of CRP

For CRP, this study detected seven possible SNPs with key functions, including rs3093058, rs3091244, rs1417938, rs1800947, rs3093066, rs1205, and rs2808630. As revealed by haplotype analysis, tri-allelic rs3091244 (G>A) was the most critical SNP. Here, we found that individuals with A and T mutant genes at locus rs3091244 might have a higher level of susceptibility tendency compared to individuals with C allele. The allele CRP rs3091244 was significantly associated with the AAA risk and the recessive models of inheritance (A/A+T/T+A/T vs A/C+T/C+C/C, *p *= 0.011), but not the dominant model of inheritance. There was statistical significance in ORs produced from the additive model (A/A+T/T+A/T vs C/C) for the homozygous model, but not for the heterozygous and allele models. However, the C allele showed correlation with the decreased CRP level, whereas the increased CRP level was associated with the A or T allele. Typically, both the rare T allele and the A allele were related to the increased CRP level. The results on the T and A alleles have been verified by many articles [12, 65, 66]. More importantly, the genetic variant tri-allelic rs3091244 has been verified for its function, which showed different allelic frequency in the European population compared to the Asian population [67]. This might explain the reason why the CRP level had an impact on the European and Oceanian populations, while the CRP level in Asians had no effect on the susceptibility to AAA.

Simultaneously, the CRP gene locus rs1800947 was under the allele model (G vs C) and the heterozygous model (G/C vs C/C). There was no difference in the susceptibility to AAA between the GC genotype population and the CC genotype population, or between individuals carrying the G mutant gene and those carrying the C allele of rs1800947. There was no difference in the susceptibility to AAA, and the CRP locus rs1205 was under the dominant gene model (T/T+T/C vs C/C). In addition, difference in the susceptibility to AAA was not significant between the TT, TC and CC genotypes.

#### 4.2.2 SNPs of ILs

ILs are responsible for transmitting information, activating and modulating immunocytes, and mediating the growth/activation/differentiation of B and T cells. They also have key functions in inflammatory response. IL-6 is produced by aneurysm, and its expression is associated with aneurysmal surface area [52]. The circulating IL-6 levels are higher among AAA cases [52, 68]. Angiotensin II has a certain effect on AAAs by regulating the IL-6 pathway in mice.

The associations of IL-6 SNPs with AAA susceptibility have been analyzed among diverse populations [14, 69], however, no consistent results were obtained in these studies. In the present study, we found that individuals carrying the G mutant gene of rs1800795 (IL-6 gene locus) might be less susceptible to AAA than those with C allele (OR = 0.91). In addition to that, the homozygous or heterozygous recessive genotypes of IL-6/rs1800795 SNPs might not increase the AAA susceptibility. IL-6/rs1800795 SNPs were not related to AAA risk, as reported in several other studies [14, 15, 70]. Jones *et al*. [71] suggested that a mutation existed in more than one allele of -174G/C SNP, which might be related to cardiovascular mortality for patients with small aneurysms in the future.

IL-10 accounts for the potent anti-inflammatory factor, which inhibits the function of macrophages and indirectly affects T lymphocytes through regulating cell signals related to T cell antigen presentation. In this meta-analysis, individuals carrying the IL-10 locus rs1800896, the A mutant gene, might have a higher susceptibility to AAA than individuals with the G allele either in the recessive (A/A vs G/G+A/G) or the dominant (A/A+A/G vs G/G) gene model.

#### 4.2.3 SNPs of TNFs

TNF was first discovered because of its anti-cancer activity. It is now considered to coordinate the extremely complex response of the body towards injury and infection. The TNF-α-308G/A (rs1800629) SNP can be detected in chromosome 6p21.3, where the substitution of G with A results in the substitution of adenosine for guanine [72, 73]. Such alteration has direct effect on gene modulation and is related to changes in the transcriptional activity of TNF-α in numerous disorders [74]. It should be noted that the A allele of TNF-α-308 displays increased *in vitro* activity compared with the common G allele, which is related to the increased TNF-α expression [75]. However, no correlation between TNF-α/rs1800629 SNPs was found in our study, which suggested that the rs1800629 gene polymorphism had no effect on the susceptibility towards AAA.

### 4.3 Limitation

Certain limitations should be noted in the present meta-analysis. Firstly, this meta-analysis was not adjusted for correcting the commonly seen risk factors for AAA (such as smoking, age, and gender), because no consistent reports on these factors are available in any independent study. As a result, those P-values and ORs obtained might be the over-estimated true biological risks. Additionally, there were obvious inter-study differences in the control population screening approach. Among our enrolled articles, merely 4 selected the control subjects by ultrasound, while other articles selected the non-specific approaches like questionnaires or did not mention control selection at all. Therefore, certain articles might have false negative diagnoses. In this regard, the control populations might not actually represent the normal (AAA-free) individuals. Moreover, based on the statistical analysis, many articles (or many SNPs examined in the present meta-analysis) did not have adequate capacity in detecting the relations because having a small study size. Lastly, the above findings should be interpreted under the meta-analysis constraints. The cumulative results might be confounded by variable quality based on methodological, heterogeneity, and publication bias among the enrolled articles. Our analyses suggest that the above factors might not interpret the results in their entirety; nevertheless, our results should be interpreted with caution due to the relatively small number of articles enrolled in the present meta-analysis.

## 5. Conclusions 

We report a systematic review and meta-analysis on the association of inflammation mediators level and gene polymorphisms and AAA. The meta-analysis demonstrated a significant difference between C-reactive protein (CRP) and IL-6 levels in patients with and without AAA. Our analyses suggest that individuals with A and T mutant genes at locus rs3091244 (CRP) might have a higher tendency of AAA susceptibility than those with C allele. In addition, individuals with a G mutant gene at locus rs1800795 (IL-6) might be less susceptible to AAA than those with C allele. Further investigation of this marker may improve our understanding of AAA pathogenesis and benefit targeted AAA screening programs.

## Data Availability

The datasets used or analyzed during the current study are available from the corresponding author on reasonable request.

## Supplementary Material

Supplementary material associated with this article can be found, in the online version, at https://doi.org/10.31083/j.rcm2308270.
