# Supplementary material for: Circulating Inflammatory Mediators and Genetic Polymorphisms of Inflammation Mediators and Their Association with Factors Related to Abdominal Aortic Aneurysm: A Systemic Review and Meta-Analysis
Source: Rev Cardiovasc Med. 2022 Jul 25;23(8):270. doi: 10.31083/j.rcm2308270 (PMC11266967; doi:10.31083/j.rcm2308270)
Supplement: Supplementary file 1 [file 2153-8174-23-8-270-s1.doc]

**Supplementary File 1.** Search strategy。

| PubMed | | |  |
| --- | --- | --- | --- |
| Step | Retrieve records | Num |  |
| 1 | ((C-reactive protein) OR(CRP))AND(abdominal aortic aneurysm) | 256 |  |
| 2 | ((IL-6) OR (interleukin 6)) AND (abdominal aortic aneurysm) | 279 |  |
| 3 | ((IL-10) OR (interleukin 10)) AND (abdominal aortic aneurysm) | 82 |  |
| 4 | （(tumor necrosis factor α) OR (TNF-α)）AND（abdominal aortic aneurysms） | 234 |  |
| 5 | ((rs3091244)OR((CRP)AND((gene)OR(SNP))))AND(abdominal aortic aneurysm) | 10 |  |
|  |
| 6 | ((rs1800947)OR((CRP)AND((gene)OR(SNP))))AND(abdominal aortic aneurysm) | 10 |  |
| 7 | ((rs1205)OR((CRP)AND((gene)OR(SNP))))AND(abdominal aortic aneurysm) | 10 |  |
| 8 | ((rs1417938)OR((CRP)AND((gene)OR(SNP))))AND(abdominal aortic aneurysm) | 10 |  |
| 9 | ((rs1800795) OR (((IL-6) OR (interleukin 6))AND((gene)OR(SNP))))AND (abdominal aortic aneurysm) | 65 |  |
| 10 | ((rs1800896) OR (((IL-10) OR (interleukin 10))AND((gene)OR(SNP))))AND (abdominal aortic aneurysm) | 22 |  |
| 11 | ((rs1800629) OR (((TNF-α) OR (tumor necrosis factor α))AND((gene)OR(SNP))))AND (abdominal aortic aneurysm) | 68 |  |

**Supplementary File 2.** The subgroup analysis shows the OR and 95% CI of the association between CRP rs3091244 and abdominal aortic aneurysm. (A) rs3091244 dominant gene model; (B) rs3091244 recessive gene model; (C) rs3091244 homozygous model; (D) rs3091244 heterozygous model; (E) rs3091244 allele model; (F) Egger test under allele model.

**Supplementary File 3**. A subgroup analysis illustrates the SMD and 95% CI for the association between plasma interleukin-6 levels and abdominal aortic aneurysm. (A) subgroup analysis of plasma IL-6 levels; (B) sensitivity analysis of plasma IL-6 levels; (C) Egger test of plasma IL-6 levels; (D) IL-6 rs1800795 allele model Egger test.

**Supplementary File 4**. The Egger test and sensitivity analysis of plasma interleukin-10 levels and rs1800896 locus and abdominal aortic aneurysm. (A) Egger test of IL-10 level; (B) Sensitivity analysis of IL-10 levels; (C) Egger test of rs1800896; (D) Sensitivity analysis of rs1800896.

**Supplementary File 5**. The Egger test and sensitivity analysis of tumor necrosis factor alpha levels and abdominal aortic aneurysms. (A) Egger test of TNF-α level; (B) Sensitivity analysis of TNF-α level.
